# Secondary Pulmonary Vein Stenosis Due to Total Anomalous Pulmonary Venous Connection Repair in Children: Extravascular MDCT Findings

**DOI:** 10.3390/children8090726

**Published:** 2021-08-25

**Authors:** Edward Y. Lee, Sara O. Vargas, Kathy J. Jenkins, Ryan Callahan, Halley J. Park, Zachary Gauthier, Abbey J. Winant

**Affiliations:** 1 Department of Radiology, Boston Children’s Hospital and Harvard Medical School, Boston, MA 02115, USA; Halley.Park@childrens.harvard.edu (H.J.P.); Abbey.Winant@childrens.harvard.edu (A.J.W.); 2 Department of Pathology, Boston Children’s Hospital and Harvard Medical School, Boston, MA 02115, USA; Sara.Vargas@childrens.harvard.edu; 3 Department of Cardiology, Boston Children’s Hospital and Harvard Medical School, Boston, MA 02115, USA; Kathy.Jenkins@childrens.harvard.edu (K.J.J.); Ryan.callahan@cardio.chboston.org (R.C.); zack.gauthier0717@gmail.com (Z.G.)

**Keywords:** secondary pulmonary vein stenosis, total anomalous pulmonary venous connection (TAPVC), multidetector computed tomography (MDCT) angiography, mediastinal disease, children, pediatric patients

## Abstract

Purpose: To evaluate extravascular findings on thoracic MDCT angiography in secondary pulmonary vein stenosis (PVS) due to total anomalous pulmonary venous connection (TAPVC) repair in children. Materials and Methods: All patients aged ≤18 years with a known diagnosis of secondary PVS after TAPVC repair, confirmed by echocardiography, conventional angiography, and/or surgery, who underwent thoracic MDCT angiography studies between July 2008 and April 2021 were included. Two pediatric radiologists independently examined MDCT angiography studies for the presence of extravascular thoracic abnormalities in the lung, pleura, and mediastinum. The location and distribution of each abnormality (in relation to the location of PVS) were also evaluated. Interobserver agreement between the two independent pediatric radiology reviewers was studied using kappa statistics. Results: The study group consisted of 20 consecutive pediatric patients (17 males, 3 females) with secondary PVS due to TAPVC repair. Age ranged from 2 months to 8 years (mean, 16.1 months). In children with secondary PVS due to TAPVC repair, the characteristic extravascular thoracic MDCT angiography findings were ground-glass opacity (19/20; 95%), septal thickening (7/20; 35%), pleural thickening (17/20; 85%), and a poorly defined, mildly heterogeneously enhancing, non-calcified soft tissue mass (17/20; 85%) which followed the contours of affected pulmonary veins outside the lung. There was excellent interobserver kappa agreement between two independent reviewers for detecting extravascular abnormalities on thoracic MDCT angiography studies (k = 0.99). Conclusion: Our study characterizes the extravascular thoracic MDCT angiography findings in secondary pediatric PVS due to TAPVC repair. In the lungs and pleura, ground-glass opacity, interlobular septal thickening, and pleural thickening are common findings. Importantly, the presence of a mildly heterogeneously enhancing, non-calcified mediastinal soft tissue mass in the distribution of the PVS is a novel characteristic thoracic MDCT angiography finding seen in pediatric secondary PVS due to TAPVC repair.

## 1. Introduction

Pulmonary vein stenosis (PVS) is caused by a decrease in luminal caliber of one or more of the extra-pulmonary veins. PVS is mainly classified into two types: primary and secondary [1,2]. While primary PVS occurs in the absence of previous intervention, secondary PVS is known to develop following total anomalous pulmonary venous connection (TAPVC) repair with an incidence of 11–17% [1,2,3,4,5,6]. Affected pediatric patients typically present with non-specific signs and symptoms, such as shortness of breath, failure to thrive, hypoxemia, pulmonary hypertension (HTN), and fatigue [1,2,3,4,5,6]. Mainly due to its rarity and non-specific clinical presentation, PVS diagnosis is often missed or delayed, resulting in suboptimal patient care. Unfortunately, delayed diagnosis of PVS is associated with increased morbidity and mortality [3,4,5,6,7,8,9,10]. Consequently, imaging studies, which can visualize the narrowed pulmonary veins associated with PVS as well as the extravascular findings of PVS, are critical for early and accurate diagnosis [11,12,13,14].

Although echocardiography and conventional angiography have been the gold standard for the imaging evaluation of PVS, both of these modalities have limitations and disadvantages in the pediatric population [11,12,13,14]. Specifically, the image quality of echocardiography depends on the availability of an optimal acoustic window for sonographic visualization of the pulmonary veins, operator skill, and patient cooperation. Conventional angiography is unfortunately an invasive procedure with associated risks, including risk of vascular injury related to direct vascular access and complications related to sedation. Consequently, there is a need for alternative and non-invasive imaging modalities, such as multidetector computed tomography (MDCT), that can aid in accurate diagnosis of PVS as well as provide comprehensive, cross-sectional anatomic characterization of both the vascular and extravascular thoracic structures (e.g., lungs, pleura, and mediastinum) [11,12,13,14].

Although in the past, the focus of thoracic MDCT angiography evaluation of PVS has been the detection of pulmonary vein narrowing, the extravascular imaging findings of PVS are useful for suggesting the diagnosis of PVS [12,13,14]. Specifically, a recently published study aimed at investigating the extravascular thoracic MDCT angiography findings of primary pediatric PVS (without underlying cardiovascular anomalies) showed the novel finding of a mildly heterogeneously enhancing, non-calcified soft tissue mass in the distribution of PVS, which has been speculated to reflect myofibroblast-like soft tissue proliferation [14]. However, to our knowledge, there have been no prior studies specifically focused on the extravascular imaging findings of secondary PVS using thoracic MDCT angiography. Therefore, the purpose of this study is to investigate the extravascular thoracic MDCT angiography findings of secondary pediatric PVS due to TAPVC repair, which is one of most frequent causes of secondary PVS in the pediatric population.

## 2. Methods

### 2.1. Institutional Review Board Approval

The Institutional Review Board at Boston Children’s Hospital approved this retrospective study. Due to the nature of this study, limited to retrospective medical/radiologic record review, informed consent was waived.

### 2.2. Study Cohort

A computerized search of our hospital’s radiology, cardiology, and pathology departmental databases was performed in order to identify consecutive pediatric patients (≤18 years) with a diagnosis of secondary PVS due to TAPVC repair, confirmed by echocardiogram, conventional angiography, and/or surgery who underwent thoracic MDCT angiography studies between July 2008 and April 2021.

Twenty diagnostic-quality thoracic MDCT angiography studies from 20 individual pediatric patients with secondary PVS due to TAPVC repair constituted the final study population. All 20 pediatric patients included in the study group had only isolated TAPVC and subsequent surgical repair, without history of other congenital heart disease or other medical comorbidities including primary lung disease. For each patient, only the initial thoracic MDCT angiography study performed at the time of diagnosis of secondary PVS after TAPVC repair was included. The mean time interval between the date of secondary PVS diagnosis and thoracic MDCT angiography study was 6.4 months (SD: 10.5; range: 0 day to 42 months).

### 2.3. Patient’s Clinical Information

Each patient’s demographic information including age, gender, and underlying cardiac anomalies was collected.

### 2.4. Pulmonary Vein Stenosis Diagnostic Criteria

Diagnosis of PVS in pediatric patients was based on previously accepted criteria, which included pulmonary vein luminal narrowing in 2 or more vessels with a mean gradient of at least 4 mm Hg by echocardiography or conventional angiography [14,15]. Among the 20 patients in the study group, PVS was diagnosed based on echocardiography alone in 9 patients (45%), conventional angiography alone in 5 patients (25%), and both echocardiography and conventional angiography in 5 patients (25%). In the remaining 1 patient (5%), the diagnosis of PVS was made at surgery.

### 2.5. Thoracic MDCT Angiography Technique

#### 2.5.1. MDCT Scanner Types

All 20 thoracic MDCT angiography studies included in this study were obtained using one of following MDCT scanners: (1) a 16-MDCT scanner (*n* = 2; 5%); (2) a 64-MDCT scanner (*n* = 14; 70%); (3) a 96-MDCT scanner (*n* = 3; 15%); (4) a 128-MDCT scanner (*n* = 1; 5%); and (5) a 256-MDCT scanner (*n* = 1; 5%).

#### 2.5.2. Thoracic MDCT Parameters

All 20 thoracic MDCT angiography studies (100%) were obtained with intravenous (IV) contrast with a contrast dose of 1.5–2 mL/kg following institutional guidelines at the time of this study. Low-radiation-dose thoracic MDCT angiography parameters, including weight-based kilovoltage, low-dose tube current, and a high-speed mode (rotation time ≤ 1 s), were used for all 20 studies. When optimal contrast enhancement (>200 Hounsfield Unit (HU)) in the left atrium was reached on a monitoring scan, MDCT angiography from the thoracic inlet level to the diaphragm level was scanned, progressing from a cranial to caudal.

### 2.6. Thoracic MDCT Angiography Image Reconstruction and Review

#### 2.6.1. Thoracic MDCT Angiography Image Reconstruction

After obtaining the initial thoracic MDCT image dataset, thin section (submillimeter) and 2.0–2.5 mm slice thickness axial CT images were reconstructed for reviewing. In addition, two-dimensional (2D) multiplanar (e.g., coronal and sagittal) reformatted CT images were also created from the initial datasets with submillimeter slice thickness. All axial and 2D multiplanar CT images were generated in both standard lung (level, −500 Hounsfield units (HU); width, 1500 HU) and soft tissue (level, 40 HU; width, 450 HU) window settings.

#### 2.6.2. Thoracic MDCT Angiography Image Review Preparation

Prior to reviewing the thoracic MDCT angiography images, the following steps were taken to minimize potential reviewer bias: (1) all patient identifiers were removed from images; (2) thoracic MDCT angiography studies were randomized before reviewing; and (3) reviewers were blinded to all other clinical and imaging study information.

#### 2.6.3. Thoracic MDCT Angiography Image Review

Two board-certified pediatric radiologists (with 5 and 11 years of experience in pediatric thoracic MDCT angiography studies) worked independently to systematically review all thoracic MDCT angiography studies. A PACS (picture archiving and communication system) (Synapse, Fujifilm Medical Systems, Stamford, CT, USA) was used for MDCT image review. For disagreed cases between these two initial reviewers, a third radiologist, a board-certified pediatric thoracic radiologist with 20 years of experience interpreting pediatric thoracic MDCT angiography studies served as a tie-breaker, without knowledge of the initial reviewers’ discordant interpretations.

### 2.7. Thoracic MDCT Angiography Image Assessment

On thoracic MDCT angiography studies, three main anatomic compartments (lung and airway, pleura, and mediastinum) were evaluated based on previously established criteria, as described in the following subsections [14,16,17].

#### 2.7.1. Lung and Airway Evaluation

The lung and airway were systematically evaluated for the presence of: (1) ground-glass opacity (GGO); (2) consolidation; (3) nodule; (4) mass; (5) cyst, (6) interlobular septal thickening; (7) fibrosis; and (8) bronchiectasis. GGO was considered to be present when an area of hazy increased lung opacity with preserved bronchial and vascular margins was identified [16]. A diagnosis of consolidation was made when there was an area of increased opacity that obscured the margins of adjacent vessels and airway walls, with or without air bronchogram(s) [16]. A pulmonary nodule was considered to be present when there was a solid lung lesion equal to or smaller than 3 cm in diameter without air bronchogram(s) [16]. The diagnosis of pulmonary mass was made when a solid lung lesion was larger than 3 cm in diameter [16]. A cyst was considered to be present when there was a round parenchymal lucency or low-attenuation area with a thin wall (<2 mm) and a well-defined interface with adjacent normal lung [16]. A diagnosis of septal thickening was made when there was increased visibility of the pulmonary interlobular septum was present [16]. Fibrosis was considered to be present when reticular opacities and/or honeycombing were identified [16]. A diagnosis of bronchiectasis was defined as dilatation of the bronchioles [16].

#### 2.7.2. Pleural Evaluation

The pleura was systematically evaluated for the presence of pleural thickening, pleural effusion, and pneumothorax. Pleural thickening was considered to be present when there was abnormally increased thickness (>1 mm) of the pleura [17]. A diagnosis of pleural effusion was made when there was fluid within the pleural cavity [17]. Pneumothorax was defined as air contained in the pleural space.

#### 2.7.3. Mediastinal Evaluation

The mediastinum was evaluated for lymphadenopathy and masses. A diagnosis of mediastinal lymphadenopathy was made based on the presence of a mediastinal lymph node larger than 1 cm in short axis in an expected mediastinal lymph node station [14]. A mediastinal mass was considered to be present when there was an abnormal soft tissue density in the mediastinum [14]. When a mediastinal mass was present, five MDCT imaging characteristics were evaluated including (1) location in relation to the area of PVS; (2) density; (3) borders (well circumscribed vs. ill defined); (4) contrast enhancement pattern (mild vs. avid and homogeneous vs. heterogeneous); and (5) presence of associated calcifications [14].

#### 2.7.4. Main Pulmonary Artery to Ascending Aorta Ratio Evaluation

The main pulmonary artery (pulmonary trunk) to ascending aorta ratio was measured on axial CT image at the level of the bifurcation of the pulmonary artery in all 20 thoracic MDCT angiography studies.

### 2.8. Statistical Analysis

Patient age at the time of thoracic MDCT angiography study and the time interval between the dates of PVS diagnosis and thoracic MDCT angiography study were normally distributed, and therefore, expressed as the mean, standard deviation, and range. The number and percentage of abnormality were calculated based on the proportion of abnormality detected on thoracic MDCT angiography studies. Interobserver agreement between the two independent reviewers regarding thoracic MDCT angiography findings was calculated using the chance-corrected kappa coefficient with the following agreement levels: (1) <0 (no agreement); (2) 0–0.20 (slight agreement); (3) 0.21–0.40 (fair agreement); (4) 0.41–0.60 (moderate agreement); (5) 0.61–0.80 (substantial agreement); and (6) 0.81–1.0 (perfect agreement). Statistical analysis was performed using SAS/STAT version 14.1 software (SAS Institute) [18].

## 3. Results

### 3.1. Study Cohort Characteristics

The final study cohort consisted of 20 discrete pediatric patients with secondary PVS due to PVS repair. In our study population (*n* = 20), 17 patients (85%) had no additional congenital anomalies. Of the remaining three patients (15%), one patient had a moderate size patent ductus arteriosus (5%), a second patient had right lung hypoplasia (5%), and a third patient had VACTERL (vertebral defects, anal atresia, tracheo-esophageal fistula, renal anomalies, and limb anomalies) association (5%). There were 17 males (85%) and 3 females (15%) ranging in age from 2 months to 8 years (mean, 16.1 months; SD, 22.8). For each patient, a single thoracic MDCT study was included (*n* = 20). Clinical signs and symptoms in study group patients included shortness of breath (*n* = 8), pulmonary hypertension (*n* = 8), hypoxemia (*n* = 8), failure to thrive (*n* = 3), pulmonary edema (*n* = 1), recurrent pleural effusions (*n* = 1), and cardiac arrest (*n* = 1). All (20/20; 100%) patients underwent TAPVC repair. At the time of last follow-up, 5/20 (25%) of patients in the study group were deceased, all due to complications of secondary PVS.

### 3.2. Thoracic MDCT Angiography Findings

All 20 thoracic MDCT studies (100%) showed the presence of PVS. The severity of PVS is graded by the extent of pulmonary vein luminal caliber narrowing into mild (<25%), moderate (25–50%), and marked (>50%). Mild degree (<25% pulmonary vein luminal size narrowing) of PVS was seen in five thoracic MDCT studies (25%). The remaining 15 thoracic MDCT studies (75%) showed marked degree (>50% pulmonary vein luminal size narrowing) of PVS.

The extravascular imaging abnormalities (in the lung and airway, pleura, and mediastinum) seen on the included MDCT angiography studies of children with secondary PVS due to TAPVC repair are summarized in Table 1.

#### 3.2.1. Lung and Airway Abnormalities

**Figure 1:**
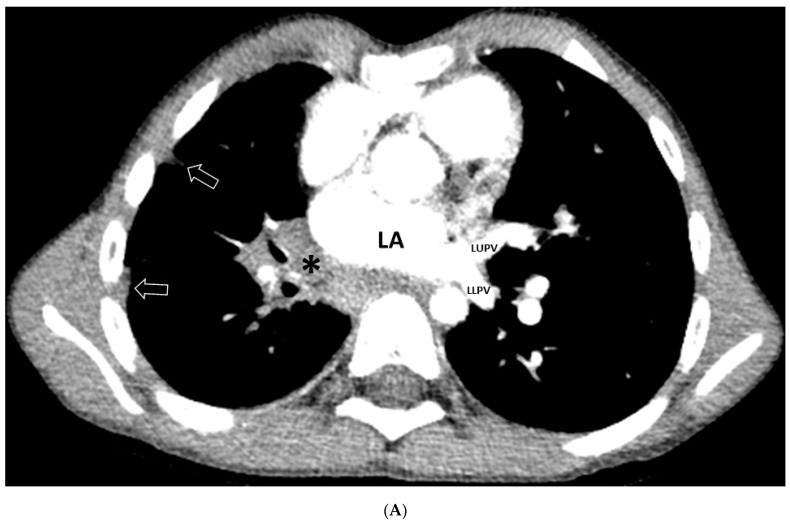

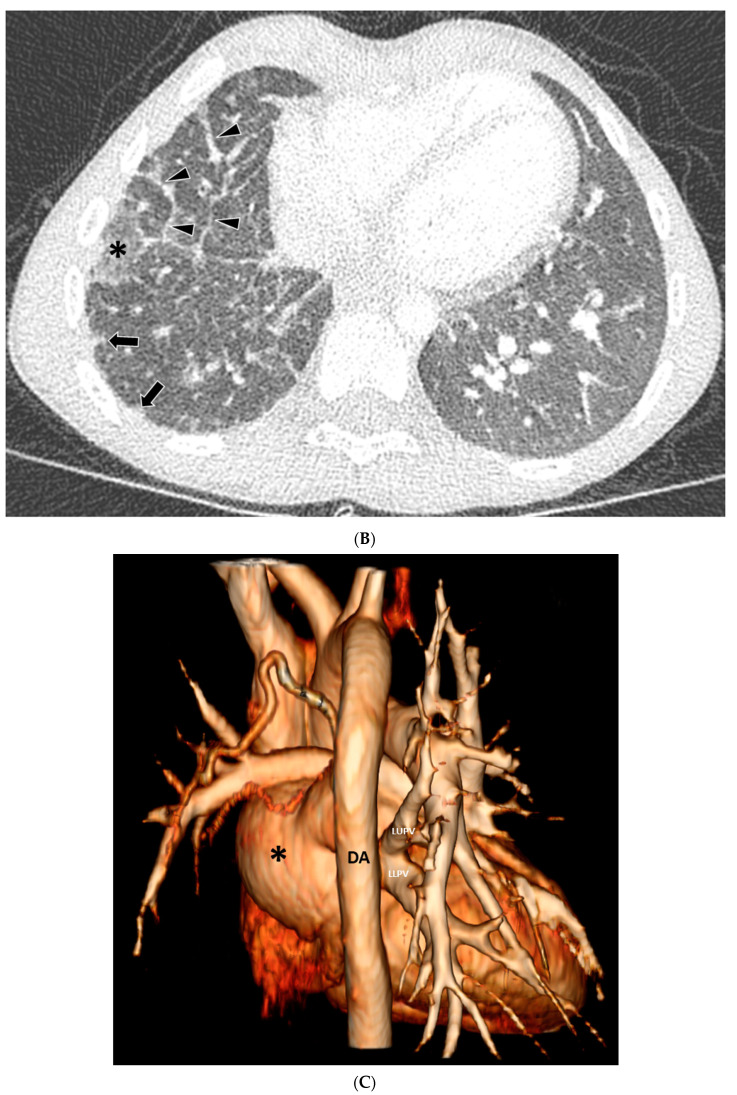
An 8-month-old male with secondary pulmonary vein stenosis due to total anomalous pulmonary venous connection repair who presented with shortness of breath. (**A**) Axial contrast-enhanced thoracic MDCT angiography CT image shows ill-defined, mildly heterogeneously enhancing, non-calcified soft tissue mass (asterisk) in the expected location of the right pulmonary vein. Right pulmonary vein is absent. Pleural thickening (arrow) is seen. Left upper pulmonary vein (LUPV) and left lower pulmonary vein (LLPV) are patent. LA = left atrium. (**B**) Axial lung window CT image demonstrates ground-glass opacity (asterisk) in the right lower lobe, septal thickening (arrowheads), and pleural thickening (arrows). (**C**) 3D volume-rendered vascular reconstruction CT image in the posterior projection view shows absent right pulmonary vein in the expected location (asterisk). Patent left upper pulmonary vein (LUPV) and left lower pulmonary vein (LLPV) are seen. DA = descending aorta.

Nineteen of twenty patients had lung abnormalities, which included GGO (19/20; 95%) and interlobular septal thickening (7/20; 35%) (Figure 1). There was no identified consolidation, nodule, mass, cyst, fibrosis or bronchiectasis. Of note, posterior-dependent atelectasis in the bilateral lower lobes was observed in two (10%) thoracic MDCT angiography studies.

#### 3.2.2. Pleural Abnormalities

Pleural abnormalities were seen in 17/20 (85%) thoracic MDCT angiography studies. The pleural abnormalities were confined to pleural thickening in all patients (Figure 1). There was no pleural effusion or pneumothorax.

#### 3.2.3. Mediastinal Abnormalities

**Figure 2:**
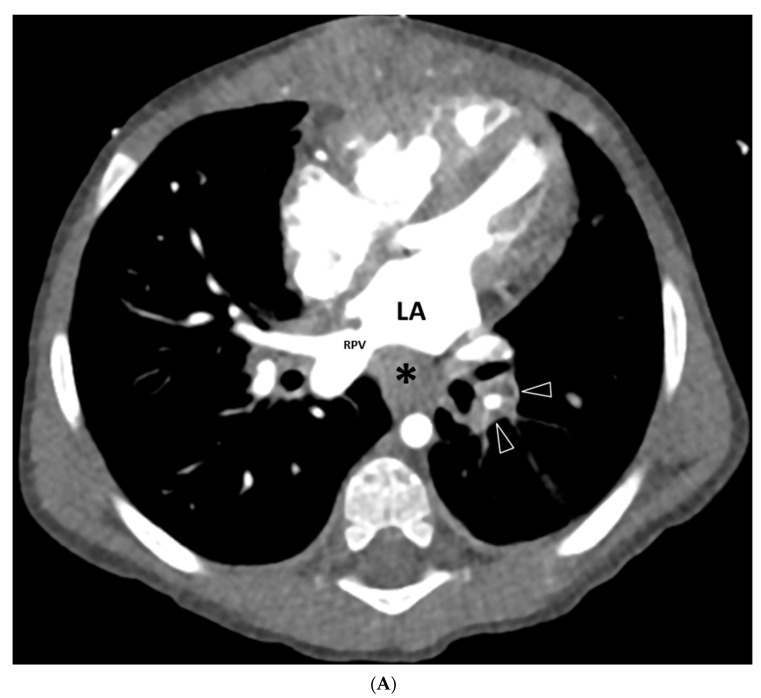

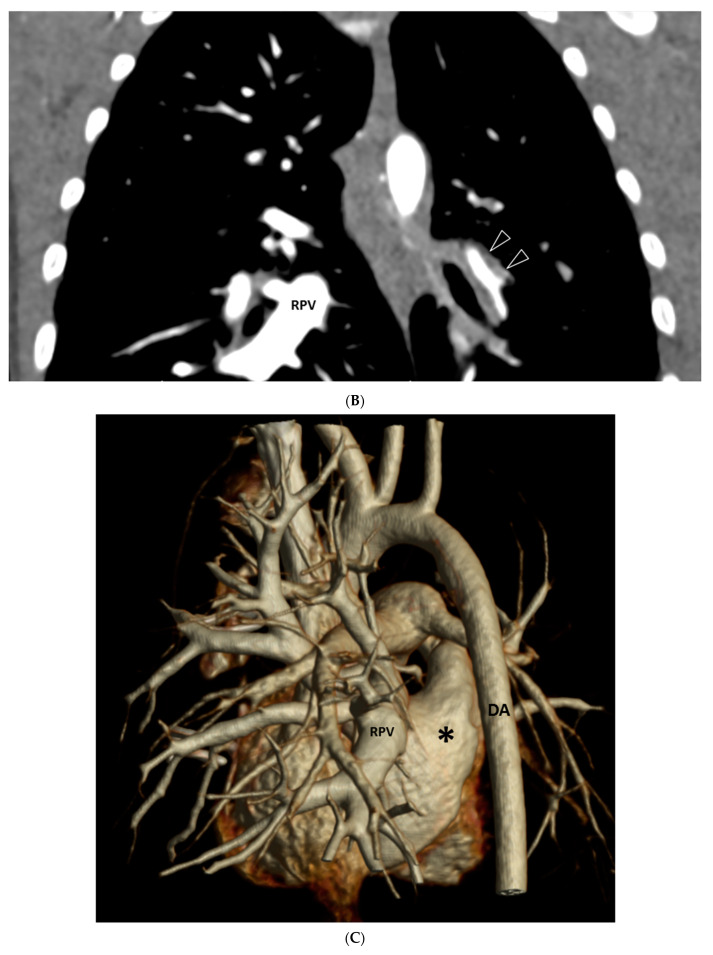

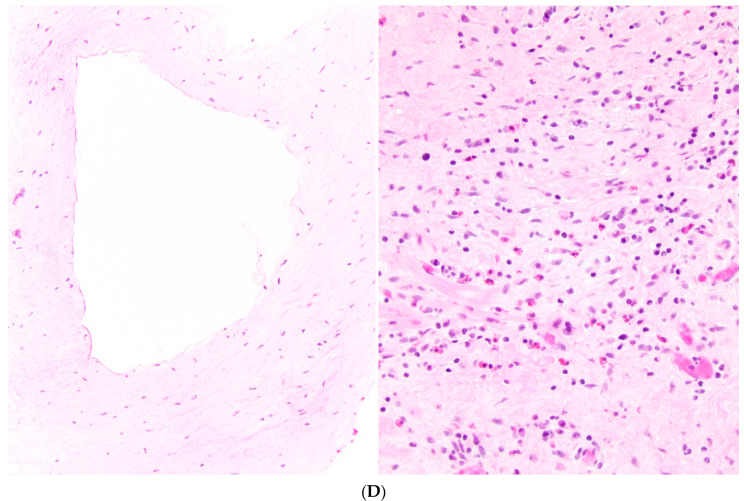
A 13-month-old male with secondary pulmonary vein stenosis due to total anomalous pulmonary venous connection repair who presented with hypoxemia. (**A**) Axial contrast-enhanced thoracic MDCT angiography CT image shows ill-defined, mildly heterogeneously enhancing, non-calcified soft tissue mass (asterisk) in the expected location of the left proximal pulmonary vein. Left proximal pulmonary vein is absent. Similar abnormal soft tissue (arrowheads) surrounding left pulmonary vein with decreased size is seen more distally. Right pulmonary vein (RPV) is patent. LA = left atrium. (**B**) Coronal contrast-enhanced thoracic MDCT angiography CT image demonstrates ill-defined, mildly heterogeneously enhancing, non-calcified soft tissue mass (arrowheads) along the narrowed left pulmonary vein. Right pulmonary vein (RPV) is patent. (**C**) 3D volume-rendered vascular reconstruction CT image in the posterior projection view shows absent left pulmonary vein in the expected location (asterisk). Patent right pulmonary vein (RPV) is seen. DA = descending aorta. (**D**) Pulmonary vein biopsy obtained at age 14 months shows circumferential intimal fibromyxoid proliferation (left panel; original magnification, 20×) and extravascular/adventitial foreign-body giant-cell reaction (not shown), fibrosis, and a cellular inflammatory infiltrate comprised of lymphocytes, plasma cells, eosinophils, and histiocytes (right panel; original magnification, 40×). Hematoxylin and eosin stain in both images.

Mediastinal abnormalities were seen in 17/20 (85%) thoracic MDCT angiography studies. All of these abnormalities were masses (*n* = 17; 85%). All masses (17/17; 100%) were ill defined; they showed mild heterogeneous enhancement and lacked calcification in all cases. Anatomically, they were distributed along the contour of veins affected by PVS, and they were entirely extravascular in their location (Figure 1 and Figure 2). Mediastinal lymphadenopathy was not identified.

#### 3.2.4. Main Pulmonary Artery to Ascending Aorta Ratio

The main pulmonary artery (pulmonary trunk) to ascending aorta ratios measured for all 20 thoracic MDCT angiography studies included in this study are summarized in Table 2. The main pulmonary artery to ascending aorta ratios ranged from 1.1 to 2.19 (mean, 1.45; SD, 0.32).

### 3.3. Interobserver Agreement

There was almost perfect interobserver kappa agreement between two independent reviewers for detecting extravascular abnormalities on thoracic MDCT angiography studies (k = 0.99). The two reviewers exhibited diagnostic concordance for all findings except on two occasions among the 20 thoracic MDCT angiography studies. Disagreement between the two reviewers was related to the presence of interlobular septal thickening in both instances. The third reviewer adjudicated the discrepancy by concluding that there was mild septal thickening in both thoracic MDCT angiography studies.

## 4. Discussion

The results of this study, which focused on the extravascular thoracic MDCT angiography findings specifically in the setting of pediatric secondary PVS due to TAPVC repair, showed the characteristic intrapulmonary findings of GGO and thickening of the interlobular septa and the pleura. In addition, for the first time, our study found the presence of a mediastinal mildly heterogeneously enhancing, non-calcified soft tissue mass following the anatomic distribution of the secondary PVS, which is a novel imaging finding detected in secondary pediatric PVS. We believe that clear knowledge of this constellation of extravascular thoracic MDCT angiography findings in secondary PVS has great potential for improving early and accurate diagnosis of PVS, even when thoracic MDCT is obtained without intravenous contrast in the pediatric population. It may also help to provide insight into the pathogenesis of PVS.

PVS is categorized into two types: primary and secondary [1,2,3,4,5]. Although the exact pathologic mechanism of these two subtypes is not clearly elucidated, comparison of imaging findings may hint as to whether there is a similar underlying mechanism. A recently published seminal study investigated, for the first time, the characteristic extravascular thoracic MDCT findings in pediatric patients with primary PVS (without underlying congenital heart disease or other medical conditions) [14]. In contrast, the primary goal of the present study was to investigate the characteristic extravascular thoracic MDCT findings in children with secondary PVS resulting from repaired TAPVC, a well-known postoperative complication. A secondary benefit of our study results is that by specifically investigating the extravascular thoracic MDCT findings of pediatric patients with secondary PVS due to TAPVC repair, it was possible to evaluate for substantial differences in the pattern of extravascular thoracic MDCT findings of primary and secondary PVS in children, in order to potentially suggest whether the underlying etiology of certain extravascular thoracic MDCT findings are congenital versus acquired.

Interestingly, our findings of lung and pleural abnormalities of secondary PVS due to TAPVC repair are similar in terms of the type and frequency when directly compared to the recently published findings seen in pediatric patients with primary PVS [14]. In our study, GGO (95%), septal thickening (35%), and pleural thickening (85%) were three characteristic lung and pleural abnormalities detected in pediatric patients with secondary PVS due to TAPVC repair. In comparison, these same three lung and pleural abnormalities (GGO (93%), septal thickening (33%), and pleural thickening (93%)) were seen with similar frequency in pediatric patients with primary PVS. Therefore, we believe that the results of our study suggest that abnormal extravascular findings on thoracic MDCT angiography are likely related to underlying PVS, regardless of whether it is primary or secondary. In regard to the underlying pathophysiological consequences of GGO, septal thickening, and pleural thickening, we agree with the previously proposed underlying causative mechanism of engorged veins in the interlobular septa and visceral pleural surface (due to obstruction of pulmonary venous connection from narrowed pulmonary veins) manifesting as GGO from alveolar (airspace) edema and alveolar septal thickening in the lungs as well as pleural thickening in the pleura on thoracic MDCT angiography [14].

In addition to lung and pleural abnormalities, our study also showed, for the first time, the presence of a mildly heterogeneously enhancing, non-calcified soft tissue mass in the distribution of PVS in the mediastinum in the vast majority of children with secondary PVS due to TAPVC repair. This abnormal mediastinal soft tissue mass in the distribution of the PVS was also seen with similar frequency (93% vs. 85%) in primary pediatric PVS MDCT angiography studies [14]. Consequently, we also believe that this abnormal mediastinal mass on thoracic MDCT angiography is specifically related to underlying PVS, regardless of whether it is primary or secondary. In the recently published radiology study focusing on primary PVS, an extravascular myofibroblastic proliferation was identified histologically; however, this has not yet been systematically studied [14]. Therefore, future study focusing on direct correlation between radiological and pathological findings of abnormal mediastinal mass in pediatric patients with PVS is needed.

Currently, there is no standardized way to screen for PVS after TAPVC repair in the pediatric population; however, patients are routinely followed with clinical assessment and echocardiogram. If all pulmonary veins cannot be completely evaluated with echocardiogram, additional non-invasive imaging studies, such as thoracic MDCT angiography, magnetic resonance imaging (MRI), and/or lung perfusion scan, are often subsequently obtained. If non-invasive imaging study findings are equivocal, further investigation can be achieved with conventional angiography and/or cardiac catheterization. Among these imaging studies, thoracic MDCT angiography is often most useful because it also provides anatomic information of all thoracic structures. The results of our study underscore the importance of evaluating all thoracic structures (including lungs, pleura, and mediastinum) in addition to the pulmonary veins when pediatric patients with suspected PVS are encountered in daily clinical practice. We believe that, due to the inherent limitations of previously considered gold-standard modalities (echocardiography and conventional angiography) for diagnosing PVS, MDCT angiography, which can provide comprehensive anatomic characterization of both the vascular and extravascular thoracic structures, is an important non-invasive imaging modality for evaluating PVS. However, we would like to emphasize that, due to potentially harmful ionizing radiation associated with MDCT, careful technical factor selection closely following the ALARA (As Low As Reasonably Achievable) is essential when MDCT angiography is considered for evaluating PVS in the pediatric population [19,20,21].

We acknowledge that there are two main limitations in our study. First, the patient population size is relatively small, which is mainly due to strict patient inclusion criteria used in our study. However, we believe that using strict patient inclusion criteria substantially improved the scientific value of our study. Future studies with larger patient populations will be helpful for confirming the results of our study. A small percentage (10%) of thoracic MDCT angiography studies included in this investigation had a small area of posterior-dependent atelectasis, limiting evaluation for lung parenchymal abnormalities. However, we believe that this did not affect the overall results of our study substantially because potential abnormal lung findings were still able to be visualized in the remaining well-aerated portion of lungs in these two studies.

In conclusion, our study, which is the first scientific investigation of the extravascular thoracic MDCT angiography findings of secondary PVS in pediatric patients due to TAPVC repair, shows characteristic extravascular thoracic MDCT angiography findings that are similar to recent findings seen in pediatric patients with primary PVS. Up-to-date knowledge of the characteristic extravascular thoracic MDCT angiography findings (i.e., GGO, septal thickening, pleural thickening, and an abnormal mediastinal soft mass around the PVS) in pediatric patients with secondary PVS has great potential to contribute to timely and accurate diagnosis, which can, in turn, lead to optimal patient care.

## Figures and Tables

**Table 1:** Summary of Extravascular Thoracic MDCT Angiography Findings of Children with Secondary PVS Due to TAPVC Repair.

Types of Extravascular Thoracic MDCT Angiography Findings	Number (Percentage) of Abnormalities (*n* = 20)
**Lung Findings**	
GGO	**19/20 (95%)**
Septal Thickening	**7/20 (35%)**
Nodule	0/0 (0%)
Mass	0/0 (0%)
Cyst	0/0 (0%)
Fibrosis	0/0 (0%)
Bronchiectasis	0/0 (0%)
**Pleural Findings**	
Pleural Thickening	**17/20 (85%)**
Pleural Effusion	0/0 (0%)
Pneumothorax	0/0 (0%)
**Mediastinal Findings**	
Mediastinal Mass	**17/20 (85%)**
Mediastinal Lymphadenopathy	0/0 (0%)

MDCT: Multidetector Computed Tomography; PVS: Pulmonary Vein Stenosis; TAPVC: Total Anomalous Pulmonary Venous Connection; *n*: Number; GGO: Ground-glass Opacity.

**Table 2:** Main Pulmonary Artery (Pulmonary Trunk) to Ascending Aorta Ratio.

Main Pulmonary Artery Size (mm)	Aorta Size (mm)	P/A Ratio
10.56	9.55	1.1
19.09	13.42	1.42
18.44	13.72	1.34
14.25	12.53	1.14
10.30	8.47	1.22
11.51	9.25	1.24
16.00	10.97	1.46
15.96	10.4	1.53
14.35	10.62	1.35
13.81	7.84	1.76
10.03	5.42	1.85
19.99	11.62	1.72
21.22	16.20	1.31
10.91	4.97	2.19
9.70	4.48	2.17
16.13	12.42	1.30
10.98	9.51	1.15
10.03	8.14	1.23
17.43	12.69	1.37
16.11	14.18	1.14

P/A = main pulmonary artery (pulmonary trunk) to ascending aorta ratio.

## Data Availability

Not applicable.
